# Infrastructure as Environmental Health Policy: Lessons from the Clean School Bus Program’s Challenges and Innovations

**DOI:** 10.3390/ijerph22081232

**Published:** 2025-08-07

**Authors:** Uchenna Osia, Bethany B. Cutts, Kristi Pullen Fedinick, Kofi Boone

**Affiliations:** 1 Center for Geospatial Analytics, North Carolina State University, Raleigh, NC 27695, USA; unosia@ncsu.edu; 2 Department of Parks, Recreation and Tourism Management, North Carolina State University, Raleigh, NC 27695, USA; 3 Milken Institute School of Public Health, George Washington University, Washington, DC 20052, USA; 4 Department of Landscape Architecture and Environmental Planning, North Carolina State University, Raleigh, NC 27695, USA

**Keywords:** environmental investment, dasymetric mapping, data-driven policy, Clean School Bus Program, federal grant programs, public funding, environmental health

## Abstract

This study evaluates the 2022 rollout of the Clean School Bus Rebate Program (CSBRP) to understand how eligibility rules and data practices shape funding distribution across communities with varying needs. We ask whether more accurate maps can improve environmental funding outcomes or whether challenges stem from how agencies define and apply eligibility criteria. Using logistic regression and dasymetric mapping, we find that prioritization criteria helped direct funds to underserved areas, but reliance on school district boundaries introduced inconsistencies that affected program reach. Including charter schools as independent applicants increased competition and sometimes diverted funds from larger public systems serving more. Our geospatial analysis shows that while refined mapping approaches improve resource targeting and reduce goal-outcome mismatches, agency discretion and administrative rules remain key factors in ensuring equitable outcomes.

## 1. Introduction

Across the United States, climate policy and environmental investments increasingly aim to correct long-standing inequities in pollution exposure and health outcomes. Yet, ensuring that these investments reach the most affected communities remains a critical challenge in both policy design and implementation. In 2021, President Biden’s Executive Order 14008, known as Justice40, set a target to deliver 40% of federal environmental benefits to disadvantaged communities [1]. Agencies developed new tools and methods for operationalizing environmental justice (EJ), culminating in tools like the Climate and Economic Justice Screening Tool (CEJST) [2]. However, questions remain about whether data and tools—and the agency discretion guiding how they are used—effectively translate federal priorities into real-world outcomes.

Harrison’s From the Inside Out underscores that meaningful EJ reform depends not only on data but also on whether agency staff see justice work, as aligned with their mission [3]. This tension between data-driven targeting and the discretionary power of program staff remains central in evaluating early Justice40 implementation. Although just a moment in time, the initial interpretation of Justice40 provides deep insight into public administration efficiency [4]. The interaction between quantitative metrics and bureaucratic discretion reveals both opportunities and constraints in translating environmental justice commitments into practical outcomes across diverse communities [3,5,6].

This study contributes to a better understanding of data use and administrator discretion in reaching policy priorities and evaluates the Clean School Bus Program’s early implementation. The EPA’s Clean School Bus Rebate Program (CSBRP) is one of the flagship Justice40 initiatives. We test the hypothesis that the program’s initial reliance on school district boundaries and simple prioritization criteria led to funding patterns that align with federal EJ goals. Specifically, we evaluate whether more refined spatial methods, such as dasymetric mapping, would yield more accurate targeting of disadvantaged communities. Our analysis contributes to ongoing debates about whether environmental justice policy outcomes are limited more by a need for sophisticated data or by the discretionary practices of federal agencies. Although a complete review is beyond the scope of this paper, environmental justice initiatives increasingly rely on spatial data tools to guide equitable investment; recent scholarship has raised important questions about the design, limitations, and governance of these tools across federal, state, and local contexts [7,8,9,10].

Our findings suggest that while the CSBRP broadly directed funds toward prioritized communities, significant mismatches arose due to boundary choices and definitional loopholes, particularly in cases where charter schools or administrative quirks skewed resource allocation. Dasymetric mapping revealed the potential to use self-certification procedures or more sophisticated mapping tools to better match intent and impact at a broad scale and to identify communities where large mismatches might suggest a need for deeper community engagement in program design. This case study offers broader insights for scholars and policymakers seeking to advance environmental justice in practice.

### 1.1. The 2022 Roll-Out of the Clean School Bus Rebate Program

**Figure 1:**
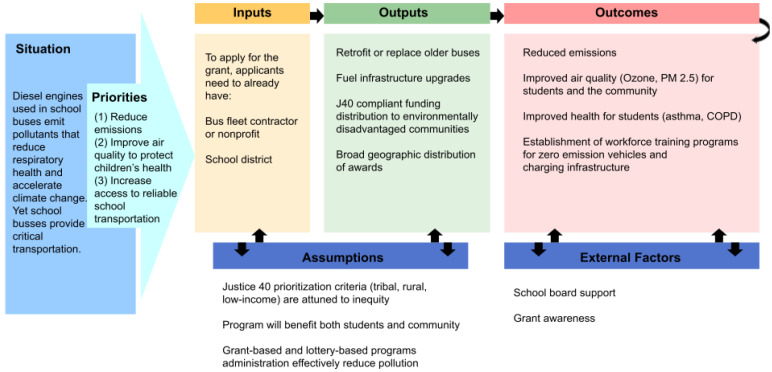
Logic model summarizing the theory of change enacted by the United States Environmental Protection Agency Clean School Bus Rebate Program (CSRBP). The logic model uses the following acronyms: J40—Justice40; PM—particulate matter; COPD—chronic obstructive pulmonary disease.

This paper examines the 2022 Clean School Bus Rebate Program, an initiative using federal infrastructure funds to reduce diesel emissions by replacing older school buses with electric or lower-emission models. Alongside fleet upgrades, the program supports charging infrastructure and workforce training to ensure long-term sustainability. By cutting harmful emissions, the CSBRP aims to address climate change, improve local air quality, and protect student and community health. This is summarized in Figure 1 as a logic model.

**Figure 2:**
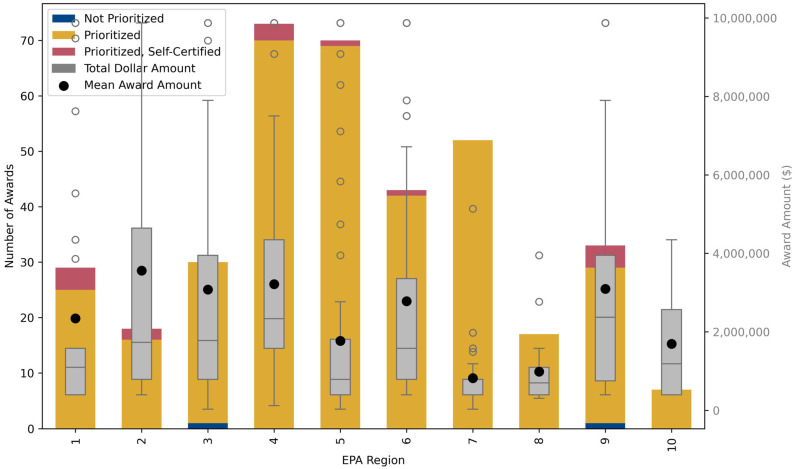
Percentage of total funds allocated to each of the ten EPA Regions through the 2022 Clean School Bus Rebate Program, showcasing regional variations in funding distribution.

**Figure 3:**
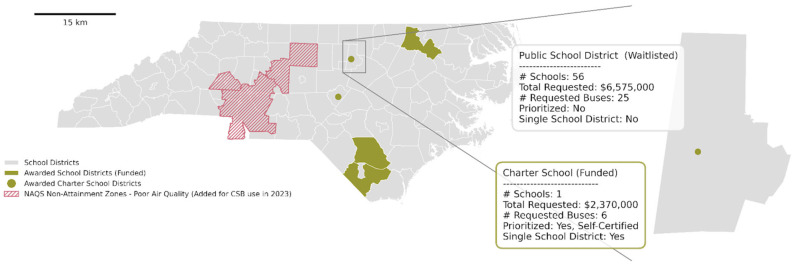
Illustration of the 2022 Clean School Bus Rebate Program and the ways that the boundary choice and data limitations shape the impact of the criteria. In the illustrated example above, selecting a boundary based on the school district leads to replacing buses for a single charter school rather than for the larger public school district, which serves a larger overall population.

In 2022, funds were distributed across the EPA’s ten Regions, with Regions 4 and 5 leading in funded districts (Figure 2) [11]. Over 1900 applications sought nearly $4 billion, though awards prioritized districts meeting eligibility criteria based on National Center for Education Statistics (NCES) data. Notably, charter schools were allowed to apply as independent districts, broadening eligibility but also potentially diverting funds from larger public districts. For example, in North Carolina, individual charter schools with a few hundred students received funding over larger districts serving tens of thousands and areas with documented air quality concerns (Figure 3). This reveals a misalignment between funding awards and environmental health risks, partly due to differences in how air quality and school district boundaries are defined.

These boundary and data issues exemplify the Modifiable Areal Unit Problem (MAUP), where changing geographic units alters program outcomes [12]. For instance, in education funding, Cheeks [13] shows that when school district boundaries are drawn to exclude or concentrate low-income populations, it can distort funding levels and obscure achievement gaps—highlighting the zoning effect of MAUP. Similarly, in transportation planning, Javanmard et al. [14] demonstrate that varying the scale or configuration of neighborhood boundaries can influence assessments of transit reliability, potentially masking the needs of underserved areas. In both cases, boundary decisions directly shape perceptions of need and influence the equity of policy interventions. Dasymetric mapping offers a solution by using land use and population data to more accurately reflect community needs. By adopting such methods, agencies could better align funding with real-world environmental and health vulnerabilities, strengthening the program’s impact.

### 1.2. Objectives

This study aims to assess the Clean School Bus Rebate Program in terms of its effectiveness and alignment with environmental justice goals. Specifically, we seek to evaluate: (1) how the program’s initial prioritization criteria impact the equitable distribution of funding to disadvantaged communities and (2) how alternative data boundaries, such as those derived from dasymetric mapping, could potentially improve the program’s strategic impact compared to traditional school district boundaries.

To achieve these objectives, we analyze CSBRP funding outcomes in four states using two complementary approaches. First, we perform a logistic regression analysis to assess whether the program’s prioritization criteria, such as rural, low-income, tribal district characteristics, and self-certification, effectively direct resources to disadvantaged communities. We include EPA Region as a random effect to account for regional funding and leadership variations. Second, we apply dasymetric mapping techniques to redistribute school district populations into finer-scale geographies, including Voronoi polygons and census blocks, to refine our spatial analysis. This combined approach allows us to evaluate whether the program’s initial geographic and funding decisions align with environmental justice goals, especially in the 2022 program cycle. The 2022 CSBRP implementation is an indicator of program discretion. It established a precedent for future program decisions to adopt a competitive grant process and came before the launch of the CEJST for use across federal programs in 2023. Ultimately, our analysis aims to determine whether more sophisticated geospatial tools could enhance the program’s capacity to meet environmental justice objectives, or whether reliance on existing administrative boundaries may distort its intended impact.

## 2. Materials and Methods

### 2.1. Study Areas

**Figure 4:**
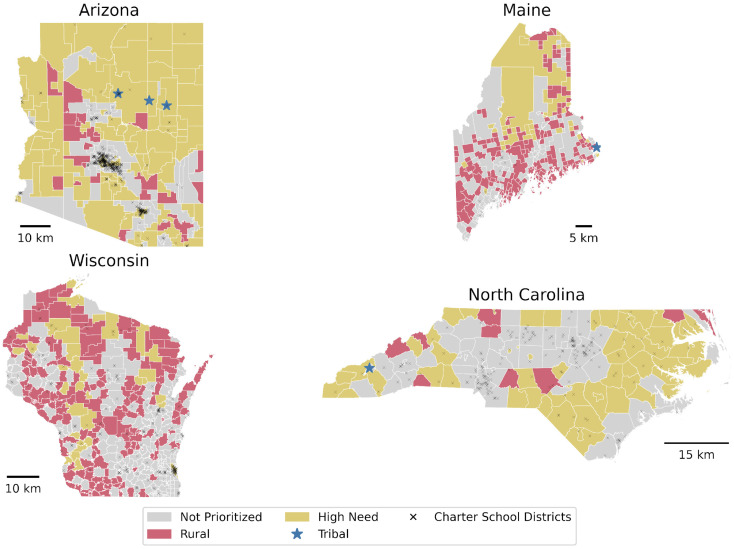
Distribution of Low-Income, Tribal, and Rural School Districts Across Four U.S. States. Data sourced from the U.S. Environmental Protection Agency (EPA) and National Center for Education Statistics Education Demographic and Geographic Estimates (NCES EDGE).

For this study, we examined EPA funding in four US States: Arizona, Maine, North Carolina, and Wisconsin. These states are situated in different EPA Regions but received similar levels of funding in 2022. Arizona received $11.8 M with 9 awarded school districts. Maine received $12.5 M with 12 awarded school districts. North Carolina received $12.2 M with 5 awarded school districts. Wisconsin received $12 M with 16 awarded school districts. Despite similar funding levels, the four US States have different school district sizes and overall state sizes (Figure 4), with Maine being the smallest, Wisconsin being the largest by area, and North Carolina the largest by population. The study areas also demonstrate differences in the visibility of disadvantaged populations as they relate to prioritization criteria. CSBRP did not include any air quality data in the initial data collection for prioritization criteria in 2022. The study data reflects the initial implementation of these dasymetric mapping techniques within our policy framework beginning in late 2022, with full adoption taking place throughout 2023.

### 2.2. Data Acquisition and Preprocessing

This study analyzed the geographic and socioeconomic distribution of awards made under CSBRP during its 2022 rollout, with attention to the program’s alignment with federal Justice40 Initiative criteria. Award data, including funded districts, award amounts, and EPA prioritization status, were obtained from the EPA’s publicly released program datasets [11]. EPA prioritization status, indicating whether awardees qualified under criteria such as serving low-income, rural, or tribal communities, was used as a proxy for Justice40 targeting.

School district boundary shapefiles were sourced from the NCES Education Demographic and Geographic Estimates (EDGE) for the 2020–2021 school year [15]. To characterize district-level socioeconomic and environmental conditions, we integrated multiple publicly available datasets. 2020 Small Area Income and Poverty Estimates (SAIPE) were drawn from the Census Bureau [16]. Air quality data was acquired from EPA’s Green Book [17]. Spatial boundaries for census block groups are from the U.S. Census Bureau’s TIGER/Line shapefiles [18].

We applied a dasymetric mapping approach to redistribute school districts and environmental attributes into census block geographies. This method, established in environmental justice and health disparities research, enables finer-scale estimation of population characteristics across alternative boundaries [19,20,21,22,23]. All spatial analyses and map generation were conducted using QGIS version 3.28 and Python version 3.12.1. The dasymetric mapping procedure followed established protocols [20] with custom scripts developed in Python.

Award patterns were then compared against district-level indicators to assess whether EPA prioritization designations correspond with areas experiencing environmental and socioeconomic disadvantage, as defined under Justice40 goals.

This study did not require human subjects research approval, as it utilized only aggregated, publicly available data.

### 2.3. Logistic Regression Analysis

If initial prioritization criteria influence the distribution of Clean School Bus Rebate Program funding to disadvantaged communities, then preliminary logistic regression results should show that school districts meeting one or more criteria, such as self-certification, rural classification, and tribal affiliation, will have a higher probability of receiving funding after controlling for potential differences across EPA regions.

To assess whether prioritization criteria effectively met the EPA’s goals of increasing the likelihood that CSBRP awards would reach disadvantaged communities or those with high air pollution burdens, we conducted a binomial logistic regression analysis. In our case, the 2022 CSBRP data indicated whether submitted applications were funded (yes) or waitlisted (no) [23]. Although some applications may have been denied outright and a few awarded projects withdrew, this information was not released.

We examined the beta coefficients across three prioritization categories to predict the likelihood of a district receiving an award: full prioritization or self-certification. Full prioritization includes districts that qualify as rural, low-income, or tribal in combination. Self-certification applies to school districts not listed in the SAIPE data used for prioritization. These schools, primarily charter schools, are eligible to self-certify when they can demonstrate having 20% or more of their students living in poverty according to the federal poverty threshold or school districts located in US territories.

If demographic prioritization is enhancing grantmaking in disadvantaged communities (in other words, if the EPA’s program is meeting its stated goal), then the regression coefficients for the prioritization criteria should be positive and statistically significant, indicating that prioritization increases the odds of receiving funding. This method is appropriate for evaluating binary policy outcomes (funded vs. not funded) given its ability to handle both categorical and continuous predictors.

### 2.4. Dasymetric Mapping

The key contribution of dasymetric mapping is not just “better maps,” but rather the improved accountability it provides for achieving complex policy goals [19]. In the case of CSBRP, which uses intricate prioritization criteria to distribute rebates, dasymetric mapping enables a more nuanced evaluation of the program’s effectiveness. By incorporating multiple data sources, it allows for a deeper understanding of how well the program is targeting communities most in need. This technique is particularly useful for identifying underserved populations that may have been missed or misrepresented in initial policy assessments based on simpler spatial boundaries [20,24].

**Figure 5:**
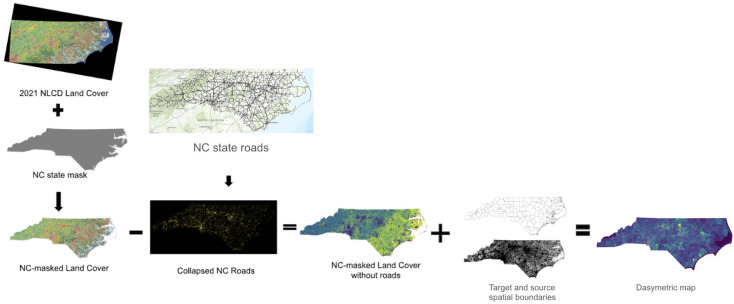
Example of dasymetric mapping process as illustrated for North Carolina USA.

In the CSBRP context, the dasymetric method allows for a more accurate representation of where environmental justice needs are most pressing, ensuring that program outcomes are not an artifact of imprecise priorities or the geopolitics of school district designations within or across states. By refining the geographic boundaries of prioritization and incorporating complex data layers, dasymetric mapping enhances the accountability of the program’s implementation and supports more equitable policy outcomes [20,21,25,26]. The dasymetric mapping procedure followed a multi-step workflow (Figure 5). First, we defined the spatial extent of school districts and census blocks using NCES EDGE and TIGER/Line shapefiles. Next, we used National Land Cover Database (NLCD) 2021 [27] land cover data to identify and remove uninhabited areas (e.g., open water, forest, industrial zones). We then constructed Voronoi polygons centered on public and charter school locations using geographic coordinates. Population from school districts was then proportionally redistributed into intersecting census blocks and Voronoi polygons based on area overlap, using the Tobler package in PySAL. To estimate population coverage for each output unit, we performed spatial intersections and applied area-weighted interpolation, assigning higher weights to blocks or polygons with larger overlaps. All spatial operations were conducted in Python using GeoPandas and GRASS GIS for raster alignment.

Due to CSBRP’s complex prioritization criteria, we employ dasymetric mapping to better evaluate the program’s effectiveness. First, we establish the program’s geographical boundaries for 2022 CSBRP prioritization. To improve the spatial precision of population and air quality data, we screen out regions unlikely to include housing using 2021 Land Cover data from the NLCD and road information from the Census Bureau [18]. Details of the data preparation using Geographic Resources Analysis Support System (GRASS GIS) version 8.2.0 and Python are provided in Figure 5.

Next, we created dasymetric maps using Voronoi polygon and census blocks boundaries. We used the Pysal Tobler package in Python. This method incorporates multiple data layers: a source variable, target variables, and ancillary data. The source variable is the population count for each school district defined within each state. The target boundaries are Voronoi polygons and Census blocks. Voronoi polygons are spatial regions defined around a set of points, where each region contains all locations that are closer to its associated point than to any other, effectively partitioning the space based on proximity. In this case, the Voronoi polygons use school locations and student populations. Census block population density refers to the number of people living within a specific census block, providing a measure of population concentration. Voronoi polygons can help analyze the proximity of a population to an air pollution source (like idling school buses) by delineating areas based on the closest distances to the source. Census blocks provide opportunities to understand and estimate how neighborhoods surrounding schools may be exposed to pollution from idling buses.

### 2.5. Error

Checking the error of dasymetric mapping model results determines how well the model represents population distribution, thereby informing reliable policy decisions. This evaluation allows for comparative analysis between different techniques, helping to identify the most effective methods for specific contexts. Furthermore, transparency in error assessment enhances the credibility of findings, making them more trustworthy. The following equations were used for Normalized Root Mean Squared Error (NRMSE), Mean Absolute Error (MAE), and Coefficient of Determination (R^2^).

(1) $$\mathrm{NRMSE}=\frac{\mathrm{RMSE}}{{\underline{y}}_{\mathrm{expected}}}$$

(2) $$\mathrm{MAE}=\frac{1}{n}\sum_{i=1}^{n}\left|\left(y_{\mathrm{expected},i}-y_{\mathrm{estimated},i}\right)\right|$$

(3) $$R^{2}=1-\frac{\sum_{i=1}^{n}{\left(y_{\mathrm{expected},i}-y_{\mathrm{estimated},i}\right)}^{2}}{\sum_{i=1}^{n}(y_{\mathrm{expected},i}-{\underline{y}}_{\mathrm{estimated},i}{)}^{2}}$$

where *n* is the number of observations, $y_{\mathrm{expected},i}$ is the expected (observed) value for the iii-th observation, $y_{\mathrm{estimated},i}$ is the estimated (predicted) value for the iii-th observation, and ${\underline{y}}_{\mathrm{expected}}$ and ${\underline{y}}_{\mathrm{estimated}}$ are the means of the expected and estimated values.

## 3. Results

A logistic mixed-effects regression was conducted to evaluate whether rural status, low-income designation, and tribal affiliation were associated with the likelihood of receiving funding, while accounting for potential variation across EPA Regions (Table 1). The model initially included EPA Region as a random intercept; however, the variance estimate for the random effect was zero, suggesting that regional variation did not meaningfully contribute to funding outcomes. As a result, the model effectively operated as a fixed-effects logistic regression. Both rural status (β = 1.15, *p* < 0.001) and low-income status (β = 0.99, *p* < 0.001) were significantly associated with increased odds of funding. Tribal affiliation showed a positive but non-significant association (β = 0.31, *p* = 0.219). The marginal R^2^ for the model was 0.128, indicating that the fixed effects explained about 13% of the variance in funding outcomes.

Due to complete separation in the data, self-certification status could not be included in the logistic regression. All 15 applicants who self-certified were funded, and none were rejected. A separate Pearson’s Chi-squared test confirmed a strong and statistically significant association between self-certification and funding (χ^2^ = 57.74, df = 1, *p* < 0.00001), reinforcing that self-certification is a highly predictive indicator of funding under this program.

Prioritized communities received approximately 51% of the total available funding, which exceeds the 40% benchmark. This indicates that the allocation of funds was more than proportional to this policy goal and suggests a strong emphasis on addressing the needs of disadvantaged communities in the award distribution process. Of the 67% of applicants that met prioritization criteria, 29% of total applicants were both prioritized and ultimately funded. These figures highlight the program’s success in steering investments toward historically underserved communities, even though some inconsistencies in boundary selection and eligibility still constrained the full realization of equity goals.

### 3.1. Overlay Analysis and Dasymetric Mapping Results

When conventional boundaries mask the truth, dasymetric mapping strips away administrative lines to expose which specific communities bear the heaviest brunt of environmental hazards. Zooming into this community-centered view may highlight areas for program remediation that could be missed when implementing large-scale national programs. While this granular view may not be the most pragmatic for national investment, it serves as a check against oversimplified policy solutions that might perpetuate or worsen existing disparities at the local level. This community-centered view also increases the chances that a federally funded program will meet its stated goals and produce measurable positive results.

**Figure 6:**
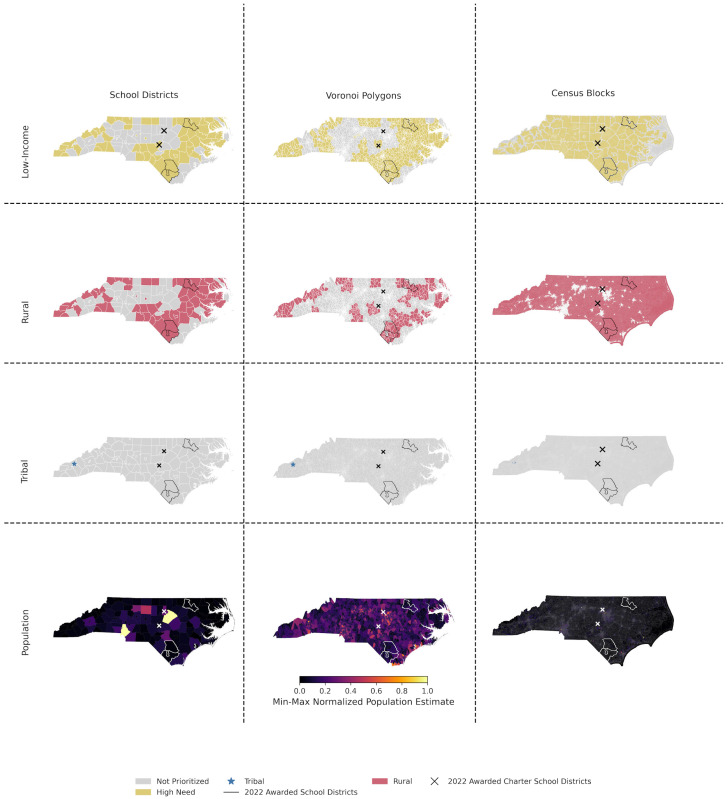
The maps utilize two dasymetric mapping techniques (Voronoi and census block population density). Maps illustrate changes in population estimates and distributions for North Carolina. Prioritization reach is also shown organized in rows, while analytical techniques are organized in columns. The maps utilize two dasymetric mapping techniques (Voronoi and block population density) and apply min-max normalization to scale the data to a fixed range of [0, 1], resulting in a linear compression of values.

The state population benefiting from CSBRP awards is the overlap in populations contained within polygon boundaries (School Districts, Voronoi, and Block Population Density). The population estimates in Figure 6 compare the total population benefitting from CSBRP funding, and it indicates that the school-district boundary is likely to underestimate the population benefitting from the CSBRP compared to estimates based on Voronoi polygons. It will also overestimate population-level benefits in relation to Block populations.

### 3.2. Error Results

The results from the block population density model were validated using U.S. Census Bureau block-level population counts (Table 2 and Table 3). To estimate population coverage within each Voronoi polygon, we applied a spatial intersection approach: block-level population polygons were intersected with Voronoi areas, and proportional populations were calculated by multiplying the share of each intersected area by its original population. Polygons with no intersection were assigned a value of zero.

Error was assessed using NRMSE, MAE, and R^2^, lower NRMSE and MAE values and higher R^2^ values indicate better performance.

Table 2 and Table 3 show that the Voronoi-based model consistently outperformed the census block model across all four states, with lower NRMSE and higher R^2^, suggesting improved fit between modeled service areas and actual population distribution. North Carolina and Maine (0.67 and 0.54, respectively) showed the strongest model fit, with R^2^ values above 0.5, indicating strong spatial congruence between school catchments and residential population areas.

By contrast, Arizona and Wisconsin exhibited higher error, particularly in NRMSE. These results align with prior findings that low-density, land-cover heterogeneous states present challenges for dasymetric disaggregation due to irregular population distribution and large spatial units [28]. However, the Voronoi model still showed substantial performance gains over the block-based approach even in these states, reinforcing Voronoi polygons utility as a ‘middle-scale’ geography that balances resolution with interpretability for policy evaluation. The overall pattern of results remains consistent across all four states: the Voronoi-based approach exhibits lower normalized error and stronger model fit (as measured by R^2^) than the census block method.

Baynes highlights that dasymetric mapping enhances resolution by excluding uninhabited areas. This improvement is crucial for public health and environmental equity applications. Extending that logic, we interpret high model error not simply as a limitation, but as a diagnostic indicator of where administrative boundaries fail to align with population reality. In this way, error flags procedural gaps in boundary logic, offering a window into policy misalignment and place-based inequity.

## 4. Discussion

### 4.1. Prioritization Criteria Improved Access

Our analysis of the 2022 Clean School Bus Rebate Program supports our central hypothesis: initial prioritization criteria play a critical role in directing federal funding to disadvantaged communities. Logistic regression results show that both full prioritization status and self-certification significantly increased the likelihood of awards reaching historically underserved areas. This finding reinforces previous research on the importance of targeted criteria in federal funding programs [29] and supports Harrison’s argument that agency staff discretion remains essential to advancing environmental protection [3]. At the same time, our results show that spatial tools not only help direct funding but also highlight where additional outreach or technical assistance may be needed to support equitable access to future opportunities. While tribal affiliation was not statistically significant in the regression model (β = 0.31, *p* = 0.219), further descriptive analysis reveals underlying patterns worth noting. Of 100 total tribal applicants, only 25 received funding, and just 6 of these were also designated as rural. This low overlap may partially explain the lack of significance in the regression model, which includes rural and low-income status as separate predictors. These findings suggest either a structural gap in how tribal applications are evaluated relative to other prioritized groups, or a possible data limitation in how tribal affiliation is recorded and integrated into prioritization scoring. Further investigation is warranted to determine whether program criteria or implementation practices contributed to the relatively low tribal award rate.

The success of the self-certification option is particularly notable. This distinction is important not only because it captures schools omitted from federal datasets, but also because it demonstrates the program’s readiness to respond to data omissions and erasures when appropriate mechanisms are in place. For example, culturally significant communities like the Gullah Geechee, an African American cultural group in coastal South Carolina, Georgia, and northeastern Florida, often have heritage centers or schools that are too small to meet the CEJST thresholds for underserved status [30]. By recognizing such gaps, the program shows both the challenges and the potential flexibility to adjust targeting criteria in response to community realities and overlooked populations. Self-certification pathways allow for recognition of place-based, community-defined disadvantages. This blend of structured and flexible criteria represents progress toward environmental health protection as a right, rather than a privilege.

Prioritization improved access to CSBRP funds, but data used to evaluate 2022 prioritization did not fully align with program goals. Although EPA has since incorporated some air quality indicators in later datasets [31], air pollution exposure remains absent from the core prioritization framework, limiting the program’s transformative potential.

### 4.2. Dasymetric Mapping Improves Allocation

Our application of dasymetric mapping provided a more precise alternative to traditional district-level allocations. By modeling schools as point sources and using Voronoi polygons to define service areas, we found that this approach produced lower errors and stronger model fit than census block models, particularly in North Carolina and Maine. These findings support the use of proximity-based spatial units in program targeting when direct exposure risk, such as from idling buses, is likely to be spatially concentrated. The CSBRP’s reliance on school district boundaries introduces the MAUP, long recognized in environmental justice research as a source of spatial inequity [11,17]. This comparative performance reinforces the potential of Voronoi polygons to act as a ‘Goldilocks scale’, more spatially precise than school districts, yet more interpretable than census blocks, for designing and evaluating equity-focused infrastructure programs. This aligns with emerging best practices in public health targeting [20,21,23] and suggests that broader adoption of such methods could mitigate both Type I errors (false positives) and Type II errors (false negatives) in funding allocations.

Yet, while promising, technical solutions alone cannot fully resolve allocation inequities. As other studies have shown [7,8,9,10], advanced mapping must be paired with programmatic reforms and meaningful community engagement to ensure that benefits reach those most impacted by environmental health risks. The dasymetric mapping method is particularly feasible for federal and state agencies seeking improved spatial precision without overhauling existing data systems. Its flexibility and interpretability make it well-suited for infrastructure programs that rely on geospatial targeting, particularly in cases where service delivery footprints do not align neatly with administrative boundaries.

### 4.3. Prioritization Must Coincide with Capacity Building

Beyond data and mapping, our findings highlight enduring procedural justice gaps that threaten the program’s ability to close environmental health disparities. Structural exclusions from transportation infrastructure and air quality protection measures continue to limit the recognition of cumulative exposures to air pollution hazards. While lack of capacity or grant-writing skills is often cited as the cause, our results suggest these gaps reflect deeper patterns of procedural exclusion rooted in historical neglect [3,32,33]. Addressing these inequities will require targeted interventions that dismantle structural barriers and expand participation pathways.

### 4.4. Limitations and Future Research Directions

This study is not without limitations. Our analysis relies on publicly available datasets, which vary in quality and granularity across states. Focusing on the 2022 program year offers an important snapshot but does not capture longitudinal trends or subsequent program adjustments. Additionally, while dasymetric mapping improves spatial targeting, further validation is needed across diverse geographic contexts. Model performance varied across states, indicating that the utility of specific dasymetric techniques may be context dependent. Future research should explore how demographic dispersion, urban-rural mix, and school siting influence the accuracy of different interpolation methods.

Future research should track CSBRP funding outcomes over multiple years, examining whether newer criteria adjustments (such as the addition of spatially refined air quality metrics) close the observed gaps. Further studies should also investigate why districts with high pollution burdens are underrepresented among awardees, and what incentives or capacity-building efforts could enhance their participation. Expanding eligibility frameworks to include cumulative burden metrics, similar to those incorporated in tools like California’s CalEnviroScreen [8], or incorporating sophisticated air pollution downscaling [34] could also help bring program implementation more in line with its stated public health goals.

### 4.5. Implications Beyond the Clean School Bus Program

While this analysis centers on the CSBRP, our findings have broader relevance for environmental justice initiatives facing similar tensions between technical design, boundary definitions, and equity outcomes. Our integrated use of dasymetric analysis and procedural justice critique offers a model for evaluating and refining resource allocation in ways that better align policy intent with real-world impact. Strengthening environmental justice programs will require pairing improved data practices with community-centered approaches that confront both technical and structural inequities head-on. To this end, we acknowledge the effectiveness of CSBRP administrators in using broad eligibility tools and self-certification as an initial step, and our findings suggest that these approaches were applied in ways that enable future program adjustments.

## 5. Conclusions

Our findings show that while refined spatial methods can improve targeting, agency discretion and administrative rules remain key factors in ensuring equitable outcomes. Our analysis of the Clean School Bus Program underscores a crucial insight for public administrators managing environmental infrastructure programs. Prioritization criteria and boundary definitions are not mere technical details but rather decisive levers that shape who benefits and who remains excluded. While the CSBRP made important strides by targeting communities facing both gaps in transportation and health infrastructure through explicit criteria and flexible self-certification pathways, persistent gaps remain, particularly in reaching areas with the greatest environmental health burdens. For infrastructure programs without an explicit Justice40 mandate, our findings offer actionable lessons. First, integrating cumulative environmental burden metrics like air pollution exposure into funding criteria can better align investments with public health goals. Second, moving beyond traditional administrative boundaries through dasymetric mapping or similar methods can mitigate inequities produced by the MAUP. Third, these more refined tools can be used to strengthen procedural fairness by identifying regions that may face high participation barriers for structurally excluded communities, including those facing capacity challenges or historical neglect. This suggests that although this study examines a single program, the diagnostic workflow it presents is broadly applicable. Program officers across other Justice40 initiatives, such as Solar for All [35] and Neighborhood Access and Equity, can adopt this approach to assess equity impacts and support more inclusive implementation.

Public administrators and agency program staff play a pivotal role as environmental health contributors. By refining allocation frameworks and expanding participation pathways, they can ensure that federal and state infrastructure investments do not merely modernize physical assets but also advance environmental justice and protect community health. As new waves of funding flow through climate and infrastructure initiatives, incorporating these lessons will be essential to transforming short-term projects into long-term public health gains.

## Figures and Tables

**Table 1:** Logistic Regression Results Predicting Clean School Bus Rebate Program funding based on prioritization status (Funded = Yes). Total *N* = 1918; *n* groups = 10; Marginal R^2^: 0.128/Conditional R^2^: 0.128. B = Beta; SE = Standard Error; CI = Confidence.

Variable	Fit	B	SE	Z	*p* > |z|	%CI
	0.128					
Intercept		−2.38	0.11	−20.91	<2 × 10^−16^	[−2.61, −2.16]
Rural (1 = Yes)		1.29	0.12	9.29	<2 × 10^−16^	[0.91, 1.39]
Low-income (1 = Yes)		1.11	0.12	7.96	1.69 × 10^−15^	[0.75, 1.23]
Tribal (1 = Yes)		0.38	0.25	1.23	0.219	[−0.18, 0.81]

**Table 2:** Comparative Performance of the Dasymetric Census Block Population Model Across States.

Variable	NRMSE	MAE	R^2^
Maine	0.79	19.24	0.39
Arizona	0.93	50.27	0.14
Wisconsin	0.95	24.57	0.14
North Carolina	0.82	30.19	0.4

**Table 3:** Comparative Performance of the Dasymetric Voronoi Polygon Population Model Across States.

Variable	NRMSE	MAE	R^2^
Maine	0.69	678.86	0.54
Arizona	0.84	2138.98	0.37
Wisconsin	0.74	1121.98	0.47
North Carolina	0.59	1061.27	0.67

## Data Availability

These data were derived from the following resources available in the public domain: National Center for Education Statistics. Education Demographic and Geographic Estimates (https://nces.ed.gov/programs/edge/Geographic/DistrictBoundaries) URL (accessed on 11 April 2024), U.S. Census Bureau TIGER/Line Shapefiles (https://www.census.gov/geographies/mapping-files/time-series/geo/tiger-line-file.html) URL (accessed on 27 May 2024), U.S. Census Bureau SAIPE School District Estimates for 2020 (https://www.census.gov/data/datasets/2020/demo/saipe/2020-school-districts.html) URL (accessed on 11 April 2024), U.S. Environmental Protection Agency (EPA) Green Book (https://www.epa.gov/green-book/green-book-data-download) URL (accessed on 11 April 2024),U.S. Geological Survey. National Land Cover Database (NLCD) 2021 Land Cover (https://www.mrlc.gov/data) URL (accessed on 11 April 2024).

## Abbreviations

EJ—Environmental Justice; CEJST—Climate and Economic Justice Screening Tool; NCES—National Center for Education Statistics; EPA—Environmental Protection Agency; CSBRP—Clean School Bus Rebate Program; J40—Justice40; PM—particulate matter; COPD—chronic obstructive pulmonary disease; NC—North Carolina; MAUP—Modifiable Areal Unit Problem; EDGE—Education Demographic and Geographic Estimates; MA—Massachusetts; MS—Mississippi; SAIPE—Small Area Income and Poverty Estimates; TIGER—Topologically Integrated Geographic Encoding and Referencing; NLCD—National Land Cover Database; GRASS GIS—Geographic Resources Analysis Support System Geographic Information System; NRMSE—Normalized Root Mean Squared Error; MAE—Mean Absolute Error; R^2^—Coefficient of Determination; B—Beta; SE—Standard Error; CI—Confidence.
